# P-1044. Interdisciplinary Initiative for Sustained CLABSI Reduction

**DOI:** 10.1093/ofid/ofaf695.1239

**Published:** 2026-01-11

**Authors:** Margo Leavitt, Alex Woods, Jessica R Miller, Bianca Johns, Julie Gregory, Sandra Williams, James Newton

**Affiliations:** Washington Regional Medical Center, Fayetteville, AR; Washington Regional Medical Center, Fayetteville, AR; Washington Regional Medical Center, Fayetteville, AR; Washington Regional Medical Center, Fayetteville, AR; Washington Regional Medical Center, Fayetteville, AR; Washington Regional Medical Center, Fayetteville, AR; Washington Regional Medical Center, Fayetteville, AR

## Abstract

**Background:**

Central line-associated bloodstream infections (CLABSIs) are correlated with elevated morbidity and mortality, with an estimated excess cost of more than $30,000 per event. Our 425-bed acute care hospital had a CLABSI incidence of 1.289/1,000 patient days with a standardized infection ratio (SIR) of 1.256, exceeding facility goals. Existing prevention strategies, including root cause analysis and a maintenance bundle had previously failed to impact facility-wide CLABSI rates.

**Figure:**
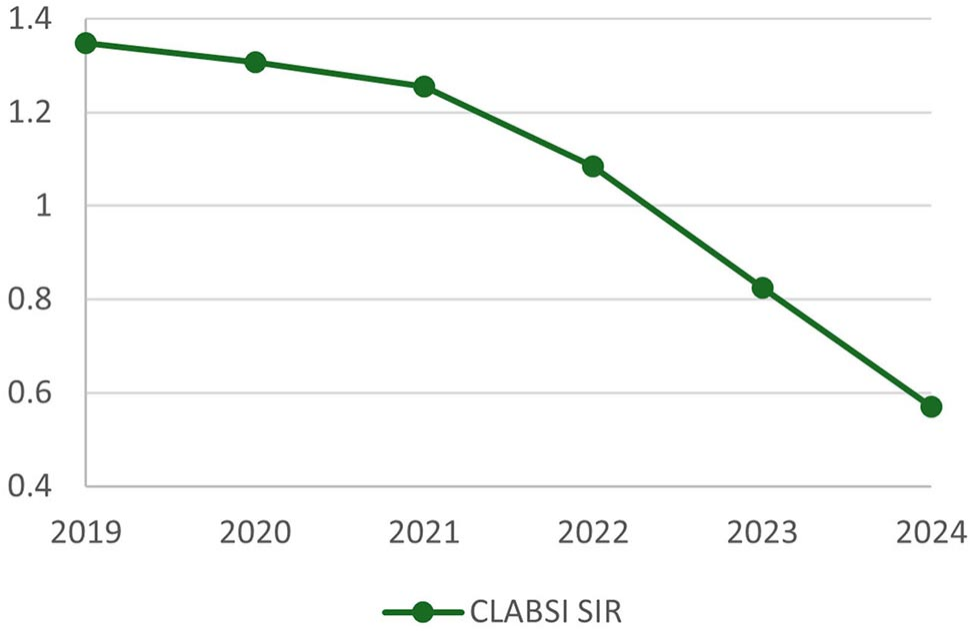

**Figure:**
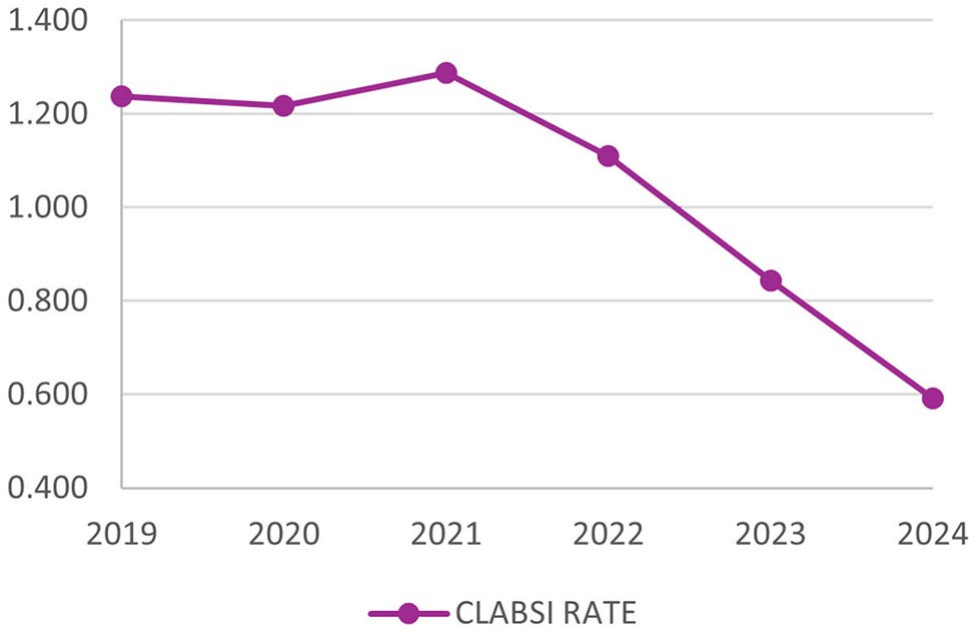

**Methods:**

Our facility initiated a collaborative CLABSI prevention quality improvement project, with dedicated ID physician and nursing champions. Areas of focus included device utilization rates, site selection, dressing maintenance, and antisepsis. The physician champion liaised with providers to establish criteria for removal and replacement of central lines in high-risk locations (femoral and internal jugular) and in CVLs placed emergently. The physician champion conducts daily audits of central line necessity in high acuity areas - critical care and oncology. In collaboration with unit leadership and bedside staff, the nurse champion conducts daily assessments of central line dressing and CHG barrier integrity. An alcoholic chlorhexidine device swab is used on all vascular access devices within high acuity areas and on each patient with a central venous catheter throughout the facility.

**Figure:**
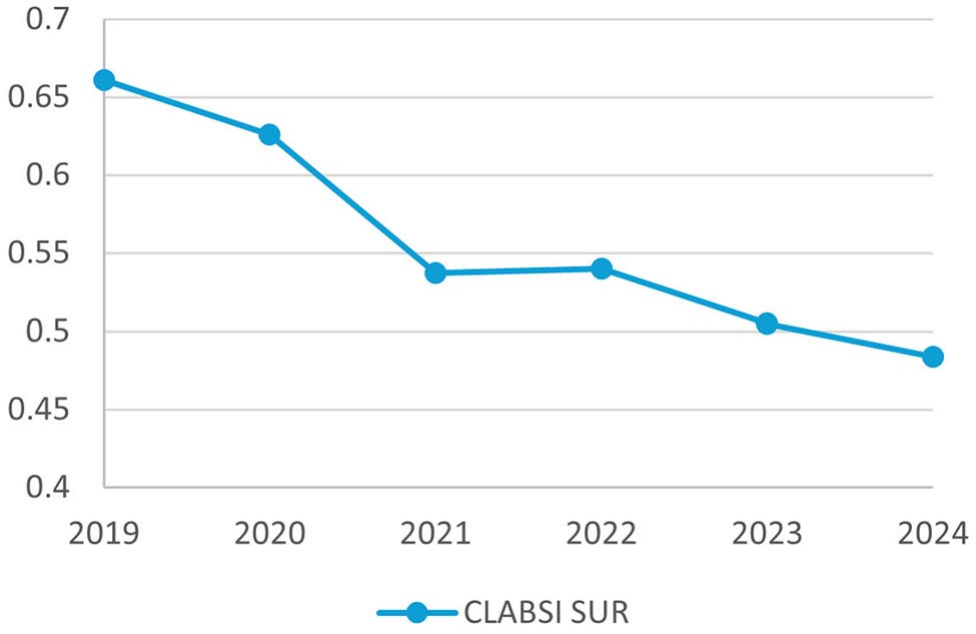

**Results:**

The project resulted in a substantial and sustained reduction in CLABSI rates, from 1.289/1,000 device days in 2021 to 0.592 in 2024. Similarly, the NHSN standardized infection ratio (SIR) decreased from 1.256 to 0.571, a 54% reduction. Furthermore, CVL utilization decreased slightly, as represented a reduction in NHSN standardized utilization ratios (SURs) from 0.537 in 2021 to 0.484 in 2024.

**Conclusion:**

By emphasizing interdisciplinary cooperation, real-time feedback, and enhanced device maintenance, our facility achieved and sustained a substantial reduction in CLABSIs. Intervention practices remain in place and our facility continues to examine processes to further integrate these practices into our culture.

**Disclosures:**

All Authors: No reported disclosures

